# Multiple Massive Bullae Formation Within Lung of SARS-CoV-2-Infected Patient

**DOI:** 10.7759/cureus.25837

**Published:** 2022-06-10

**Authors:** Sunydip Gill, Jilmil S Raina, Burak Erdinc

**Affiliations:** 1 Medicine, St. George's University School of Medicine/Brookdale University Hospital Medical Center, New York City, USA; 2 Internal Medicine, Brookdale University Hospital Medical Center, New York City, USA

**Keywords:** acute respiratory distress syndrome [ards], ards, computer tomography of chest, sars-cov-2 infection, massive bulla

## Abstract

Severe acute respiratory syndrome coronavirus 2 (SARS-CoV-2) has altered the world for more than two years and continues to impact the globe in numerous ways. This disease has a wide spectrum of presentations that can range from no symptoms at all to rapid decline and death. Complications of SARS-CoV-2 include acute respiratory failure, pneumonia, acute respiratory distress syndrome (ARDS), acute liver injury, acute cardiac injury, septic shock, blood clots, multisystem inflammatory syndrome in children, and chronic fatigue. Although many complications such as these and others exist, we are still seeing novel developments related to the virus. In this case report, we present a patient with SARS-CoV-2 who concurrently had a massive left lung bulla of unknown etiology. Due to the size and scale of this bulla, we suspect it to be a complication of his SARS-CoV-2 infection. A few cases have been described in the literature beforeand here we would like to contribute another one. Our goal is to help expand the body of evidence demonstrating the far-reaching and atypical nature that SARS-CoV-2 can present with today.

## Introduction

Symptomatic severe acute respiratory syndrome coronavirus 2 (SARS-CoV-2) primarily presents with classic lung findings of dyspnea, changes in pulmonary function testing, pneumonia-like symptoms, and fibrotic changes [1-3]. Bullae formation is a rarer presentation in relation to SARS-CoV-2.

Chest computer tomography (CT) plays a crucial role in the management of SARS-CoV-2 complications, especially in relation to pneumonia [3]. Progression of SARS-CoV-2-related pneumonia is primarily tracked via chest CT. When pneumonia presents with SARS-CoV-2, a chest CT will often initially show ground-glass opacities and consolidation. This leaves the lung with fibrocystic changes at the origins of the pulmonary insult. However, only a handful of cases go on to develop pulmonary bullae and/or pneumothorax, as reported in the literature as of this point in time [4]. 

Here we present a case of a 59-year-old man, whose SARS-CoV-2 infection was complicated by a massive lung bulla.

## Case presentation

**Figure 1:**
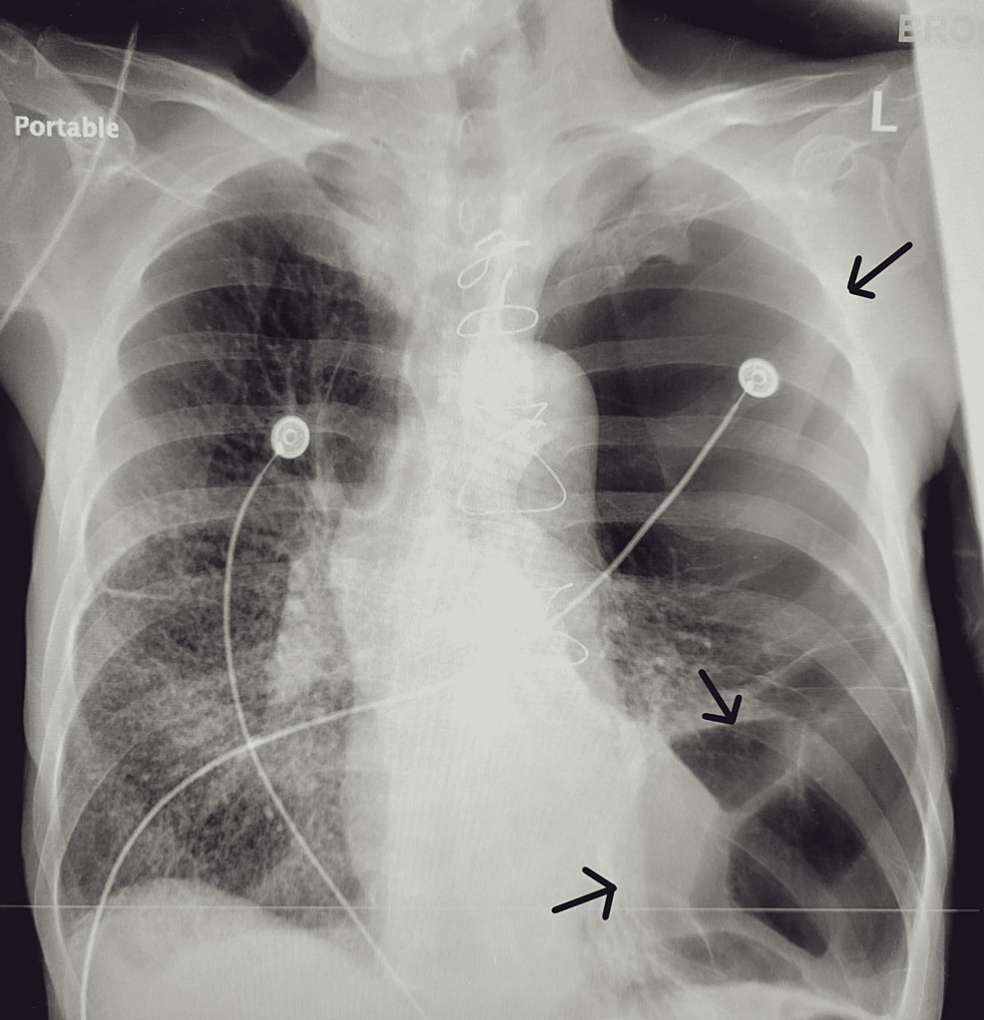
Chest x-ray: Multiple massive bullae formation (arrows) within the left lung. Moderate reticular interstitial opacities of the right lower lobe consistent with pulmonary fibrosis versus infiltrates.

We present a case of a 59-year-old man with a past medical history of thoracic surgery (whether it was lobectomy or pneumonectomy is unclear) due to gunshot wound (GSW), joint pain, and smoking cigarettes (40 packs per year). The patient presented in October 2021 with generalized weakness, abdominal pain, loss of appetite, diarrhea, and dizziness for one week. He was alert, awake, oriented to place, time, and person, and dyspneic. Physical examination revealed reduced breath sounds on the left side. On arrival, his vitals were blood pressure 94/81 mmHg, pulse 77 beats per minute, temperature 36.2 °C (97.2 °F) (oral), respiratory rate 16 breaths per minute, and body mass index (BMI) 25.77 kg/m². The oxygen saturation was 85% on room air and the patient was placed on a nonrebreather face mask. Reverse transcription-polymerase chain reaction (RT-PCR) was positive for SARS-CoV-2 at the time of admission. His chest radiograph (Figure 1) at the time of admission revealed multiple massive bullae formation throughout the left lung, completely replacing the left lower lobe. Thoracic surgery was consulted for giant pulmonary bullae.

**Figure 2:**
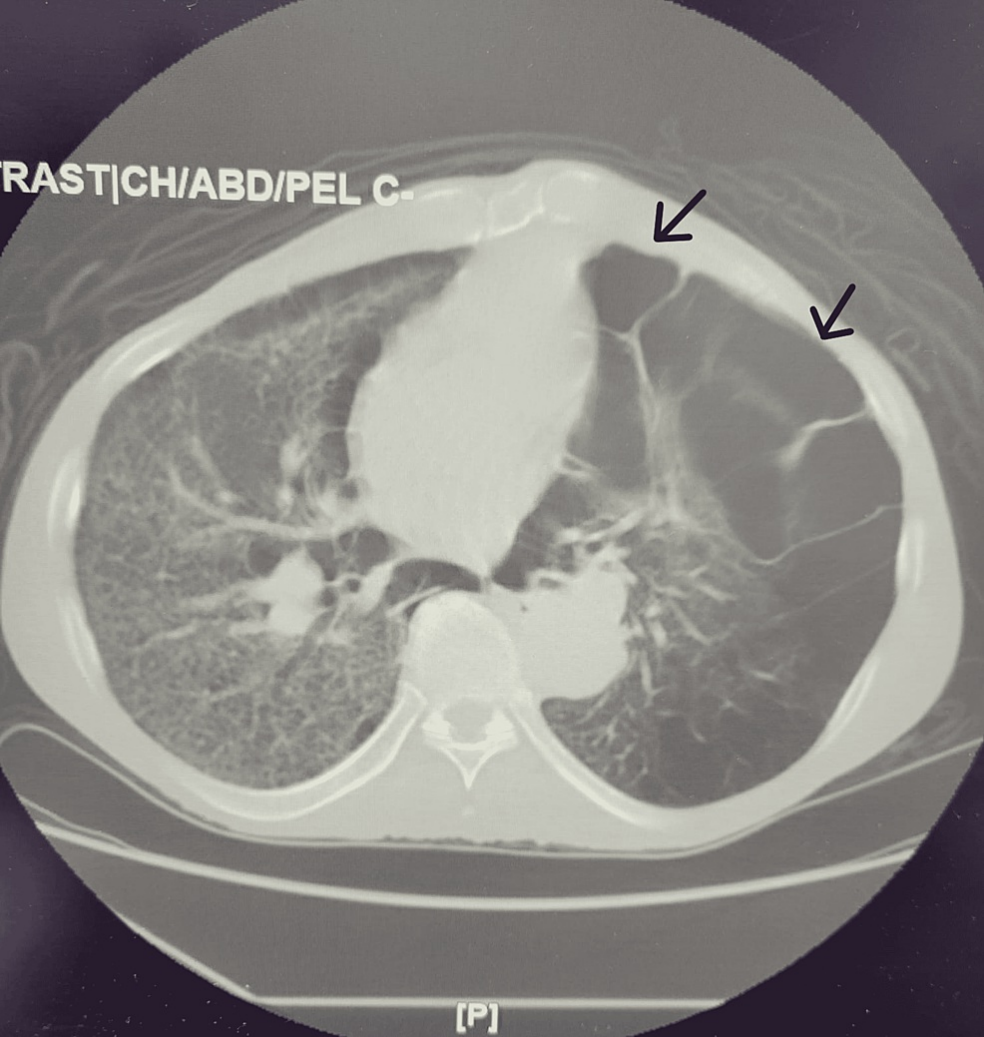
CT scan of the chest: Massive bulla within the left lung completely replacing the left upper lobe. Multiple additional large bullae (arrows) are present within the left lower lobe. Moderately severe diffuse air trapping within the right lung consistent with emphysema with fibrotic changes.

CT scan (Figure 2) showed a large bulla measuring 12 x18 x17 cm in the upper hemithorax with compression medially. There was also emphysema of the right lung. The thoracic surgeon did not recommend chest tube placement or thoracic surgery at that time. There was no relevant previous imaging to compare the current readings with.

The patient was started on high-flow nasal cannula 10 L, dexamethasone 6 mg IV, remdesivir 100 mg IV, tocilizumab 680 mg IV, ceftriaxone 1 gm, and azithromycin 500 mg IV. Due to the patient’s bleb, no other interventions were recommended at the time of the above treatments. During the hospital course, the patient refused all treatments and blood work, despite attempts made to explain to him the severity of his illness. Before treatment could be completed, the patient escaped from the hospital without medical discharge after nine days of treatment in the hospital and has yet to follow up or return to a healthcare facility.

## Discussion

This case is illustrative of one of the possible consequences of SARS-CoV-2. Although our patient was a smoker and had a history of thoracic surgery, the sheer size and impact of his bullae coupled with his lack of past imaging lead to the possibility that his SARS-CoV-2 infection could have lead to the bullae formation. There have been other such cases describing bullae associated with SARS-CoV-2 [5]. In our patient’s case, his positive SARS-CoV-2 status with his bullae adds another unique presentation to look upon as the complicated manifestations of SARS-CoV-2 are researched.* *

One of the most important screening tools for SARS-CoV-2 infection is a chest CT scan. Findings include ground-glass opacities, which are often seen in the lower lobe subpleural regions [5]. Typically, these opacities are seen in the early stages of the pneumonia and are likely caused by changes in the alveoli - whether swelling, exudate in the alveolar space, and/or septal inflammation [6].

Other lung pathologies, such as influenza and ARDS, can lead to lung bullae [7,8]. However, there have been no reports of bullae causing mass effect reported in the literature during an active SARS-CoV-2 infection. In our patient’s case, it is important to not rule out an alternative cause to his idiopathic bullae that could have coincidentally emerged during his SARS-CoV-2 infection. But with the literature demonstrating examples of lung damage pre-disposing to lung bullae, we should include our patient’s case in the body of literature exploring lesser-known impacts of SARS-CoV-2.

Unfortunately, our patient left the hospital before his treatment was completed and before additional tests and imaging could be done to explore this issue further. Our case stresses the possibility of bullae being linked with SARS-CoV-2, and also stresses the importance of management continuity while balancing the right of every patient to consent to tests and treatments. Bullae are thought to be tied to underlying pulmonary diseases such as bronchitis, emphysema, cystic fibrosis, and even cancer [9]. Our patient did have a history of thoracic surgery and hyperinflation of residual lung post lobectomy can results in bullous changes. Our patient also had a 40-year smoking history with emphysematous changes in the right lung. These could have lead to bullae formation in our patient. But the development of bullae as a complication of SARS-CoV-2 infection can not be ruled out. The development/management of bullae should be watched for and addressed when treating patients with SARS-CoV-2 infection.

## Conclusions

As SARS-CoV-2 impacts the world and burdens the healthcare system, more conditions associated with SARS-CoV-2 continue to be discovered, explored, and researched. The case reported here is an example of a less common but still important SARS-CoV-2-associated condition. Lung bullae are rarely associated with SARS-CoV-2, making each instance of bullae related to SARS-CoV-2 crucial to document. SARS-CoV-2-related lung bullae in individuals without other associated lung diseases may be an underdiagnosed entity. Further research and case studies are needed to create specific guidelines for the treatment of lung bullae secondary to SARS-CoV-2.
